# Cannibalism in temporary waters: Simulations and laboratory experiments revealed the role of spatial shape in the mosquito *Aedes albopictus*

**DOI:** 10.1371/journal.pone.0198194

**Published:** 2018-05-29

**Authors:** Valentina Mastrantonio, Graziano Crasta, Arianna Puggioli, Romeo Bellini, Sandra Urbanelli, Daniele Porretta

**Affiliations:** 1 Department of Environmental Biology, Sapienza University of Rome, Rome, Italy; 2 Department of Mathematics, Sapienza University of Rome, Rome, Italy; 3 Agriculture Environment Centre “G. Nicoli”, Crevalcore, Italy; Universita degli Studi di Camerino, ITALY

## Abstract

Cannibalism is a commonly observed phenomenon in arthropod species having relevant consequences for population dynamics and individual fitness. It is a context-dependent behaviour and an understanding of the factors affecting cannibalism rate is crucial to highlight its ecological relevance. In mosquitoes, cannibalism between larval stages has been widely documented, and the role of density, food availability and length of contact between individuals also ascertained. However, although mosquitoes can develop in temporary water habitats with very heterogeneous topologies, the role of the site shape where cannibals and victims co-occur has been instead overlooked. In this paper, we investigated this issue by using a simulation approach and laboratory cannibalism experiments between old (third- and fourth-instars) and young (first-instar) larvae of the tiger mosquito *Aedes albopictus*. Three virtual spaces with different shapes were simulated and the number of larval encounters was estimated in each one to assess whether the spatial shape affected the number of encounters between cannibal and victims. Then, experimental trials in containers with similar shapes to those used in the simulations were performed, and the cannibalism rate was estimated at 24 and 48h. Our results showed that the spatial shape plays a role on cannibalism interactions, affecting the number of encounters between individuals. Indeed, in the experimental trials performed, we observed the highest cannibalism rate in the container with the highest number of encounters predicted by the simulations. Interestingly, we found also that spatial shape can affect cannibalism not only by affecting the number of encounters, but also the number of encounters “favorable” for cannibalistic events. Temporary waters are inhabited by several species other than mosquitoes. Our results, showing an influence of the spatial shape on cannibalism in *Ae*. *albopictus* larvae, add a new critical factor to those affecting ecological interactions in these habitats.

## Introduction

Cannibalism consists of killing and eating an entire or a part of a conspecific individual [1]. This form of predator-prey interaction that occurs at intraspecific level is taxonomically spread, being observed in both vertebrate and invertebrate species [1–3].

In insects, a considerable body of work has addressed the ecological consequences of cannibalism and the factors affecting it [1–5]. Cannibalism was observed to drastically reduce population size, thus contributing to the self-regulation of populations, increasing their stability and decreasing the risk of extinction. Furthermore, cannibalism was found to increase the resilience of populations to environmental stressors, as cannibalistic individuals and those who survive to their attacks are likely to be the more vigorous individuals [5–8]. Finally, cannibalism has been shown to affect the ability of population propagules to colonize and persist in new stressful environments by affecting the dispersal, nutritional ability and development time of individuals [2,9]. Likewise, cannibalism can have some consequences also at the individual level by affecting fitness benefits and costs. Benefits include the acquisition of nutritional advantages for the cannibal that increase its development rate, survival and fertility [4,10,11], and the reduction in intraspecific competition for food, mates and other resources [4,5]. Fitness costs of cannibalistic behavior include disease transmission from victims to cannibals, the consuming of potential partners and the risk of injury by the prey [4,5]. Cannibalism is therefore recognized to be a context-dependent behavior, inducible by factors affecting the benefit-cost balance, such as the relative sizes of cannibals and victims, the quality and quantity of diet, the population density and the length of contact between cannibal and victim [1,2,4,5].

Mosquitoes include more than 3.500 species, and some of them are important vectors of human and animal diseases. The immature stages develop in temporary water habitats, where different inter- and intra-specific interactions occur, and the dynamics and the rapidly changing conditions can lead to an extreme competition between individuals, favoring cannibalistic behavior [5,12–17]. Size-dependent cannibalism has been observed in larvae of *Toxorhynchites*, *Armigeres* and *Anopheles* genus, where young first-instar larvae were cannibalized by old fourth-instar larvae [18–23]. Density and the length of contact between larvae have been also shown to affect cannibalism in several mosquito species [18,22]. Food availability has been shown to play a role as well, such as in the mosquito *Armigeres subalbatus*, where the cannibalism rate between late instars (third- and fourth-instars) was higher when food was restricted than when it was given *ad libitum* [20].

On the other hand, in some cases cannibalism has not been explained by any of the above factors, but primarily by the probability of encountering a vulnerable individual. For example, in the mosquitoes *Anopheles gambiae* s.s. and *An*. *arabiensis*, cannibalism between old fourth-instar and young first-instar larvae was increased not by reduced food quantity, but by reduced space, which suggested that larval cannibalism in these species was enhanced by more frequent encounters within smaller environments [22,23]. Likewise, the frequency of intra-instar cannibalism between larvae of the mosquito *Toxorynchites amboinensis* was smaller in short and large than in tall and thin containers [19]. From the examples above, the spatial shape of the sites where cannibals and victims co-occur seems to significantly affect the cannibalism rate by affecting the number of encounters between individuals. Spatial shape can also affect the cannibalism rate by affecting the success of cannibalistic attacks, as the escape chances of victims can differ according to location. If an encounter between the cannibal and the potential victim occurs in open water, the victim has more chance of escaping than if it occurs, for example, near the water surface or in a corner of the container [24]. Despite the fact that the spatial shape of mosquito breeding sites, where larval cannibalism occurs in nature, can be highly heterogeneous regarding dimensions and origin (e.g. small water pools, tree holes, cattle hoof prints, man-made containers), its role in the cannibalism rate between mosquito larvae remains poorly investigated. In this paper, we aimed to investigate this issue using as study system the tiger mosquito *Aedes albopictus*.

In recent decades, *Ae*. *albopictus* has spread from its native range in East Asia to Australia, Africa, Europe, and the Americas [25–30]. This species is vector of relevant animal and human diseases [29] and is common in both suburban and rural areas, where it develops in tree holes and man-made containers of any material and form, including tires and cemetery flower pots [25]. As far as we know, no studies have focused on cannibalistic interaction between larval stages of this species. Here, we aimed *i*) to assess if cannibalism occurs between old and young larval instars and *ii*) to investigate the role of spatial shape on cannibalism rate. Firstly, we used a simulation approach to estimate the number of encounters between cannibal and victims and the number of encounters weighted for the likelihood of successful cannibalism within containers of three different shapes. Then, we performed cannibalism experiments using third-, fourth- and first-instar larvae in containers with similar shapes to those used in the simulations and estimated the cannibalism rate at two time points (24 and 48 hours).

## Materials and methods

### Simulations

Three grids were created to simulate three virtual spaces represented by three parallelepipeds with volumes of about 400,000 cells. The grids simulated tall and thin, intermediate and low and wide container shapes with a fixed volume. Twenty potential victims and one cannibal (i.e. 20 first-instar and one third-/fourth-instar larvae) were assumed to be in each container. The larvae were assumed to be in a homogeneous space, initially stationaries and randomly distributed and then moving of one step/time unit. An encounter between two larvae was reported when the distance between them was below a threshold number of steps (hereafter *viewrange*). Simulations using 4, 5 and 6 threshold values were run.

Mosquito larvae belonging to the *Aedes* genus must go to the water surface to breathe through the siphon. Then, they tend to remain at the water surface and move across it to filter the bacteria and organic particles contained in the water. Furthermore, mosquito larvae tend to move toward the bottom of a container and graze on the sediments [12,31]. Therefore, on the basis of the larval behavior, we simulated the larval movement within each container as follows: each larva mostly moved toward the top and the bottom of the container; when a larva arrived at the top (or at the bottom), it moved across the surface (or the bottom), then moved toward the bottom (or the top) following a random path. The time spent in top/bottom and in the other parts of the space was set at 60 and 40%, respectively for each container on the basis of our observations of larval movements (see S1 Appendix). One million time units (i.e. 10 simulations of 100,000 steps) were simulated using the Phython programming language to compute the number of encounters between cannibals and victims (N) and their 95% confidence intervals according to the Poisson distribution.

We weighted the number of encounters for the likelihood of successful cannibalism within each container, by multiplying the estimated number of encounters between cannibal and victims (N) with the cannibalism likelihood (*p*). In each container *p* was estimated as *p* = 1 –*f*, where *f* is the space available for the victim to escape when encounters a cannibal and its value ranged from 1 to 0.125 (e.g. it is 1 when the encounter occurs in the middle of the container; 0.5 when it occurs in the top or the bottom; 0.25 in the edges and 0.125 in a corner).

### Cannibalism experiments

#### Mosquitoes

The *Aedes albopictus* mosquitoes used in this study were F2 generation derived from eggs collected by means of ovitraps during May 2016 in the urban area of Parma city (44.802 lat., 10.330 long.) and raised to adults in laboratory (no specific permissions were required for these locations/activities, and the field studies did not involve endangered or protected species). Larvae were placed into plastic trays (height = 5 cm, width = 30 cm and length = 19 cm) filled with 800 ml of distilled water, maintained in the laboratory at 27 ± 2°C, 75 ± 10% relative humidity (RH) and an L:D 16:8 h photoperiod and fed with a powder obtained by crushing dry cat food (Friskies ® Adult) (0.33g/800 ml of water) [32]. Eclosed adults were identified using the morphological keys of [33], kept in 40×40×40 cm cages and daily fed with 10% sucrose solution. Females were blood fed with fresh mechanically defibrinated bovine blood using a thermostatic apparatus [32]. Eggs were laid on paper towels in cups containing water. The paper towels were than dried and stored at 27°C until cannibalism experiments were performed. Eggs were hatched by force-hatching technique [34] to ensure uniform larval age.

#### Experimental setup

All experiments were performed under the same temperature, humidity and light/dark conditions of the reared colonies. We set up the experimental conditions at one larva/ml and no food shortage, providing food to the larvae during the experiments according to the rearing conditions described above (0.85mg/larva of fish food was supplied at the beginning of the experiment and after 24 h).

We tested the occurrence of cannibalism between third-instar (L3) and first-instar (L1) larvae and between fourth-instar (L4) and L1 larvae (L1 < 48 h old) and assessed the influence of the container shape on cannibalism rate. Three plastic containers with different surface/water column ratios were used, that reproduce common breeding sites of *Ae*. *albopictus*, such as flowerpots, man-made containers and flowerpot dishes: a 6×6×12 cm container (hereafter “tall and thin” container); a 12.5×12.5×4.5 cm container (hereafter “intermediate” container) and a 25×25×8 cm container (hereafter “low and wide” container). Each container was filled with 200 ml of distilled water, then twenty L1 larvae and one L3 or one L4 larva were placed into each container.

A control for each treatment was performed, placing 20 L1 larvae in each container without L3/L4 larvae. Further controls using dead larvae were also performed to test for possible larval decomposition and disappearance. Twenty L1 larvae (killed at -80°C for 5 min) were placed into the experimental containers maintained under the same experimental conditions as described before. Missing larvae were counted after 24 and 48 h that is the time span in which the larvae remain into their instar before moult to the next instar. For each experimental condition ten experimental containers of each shape were used (pseudoreplicates) and the experiment was replicated three times (biological replicates).

#### Data analyses

The number of L1 larvae in each container was computed after 24 and 48 h from the beginning of the experiments. Missing L1 larvae were considered cannibalized by L3 or L4 larvae [18–24]. The effect of the experimental factors and their combination on the cannibalism rate was investigated by logistic regression, using cannibalism as a binary response variable. The experimental factors used were: larval instar (L3, L4); container shape (encoded as: tall and thin = 1, low and wide = 2 and intermediate = 3); time (i.e. the length of contact between larvae, 24 and 48 hours). The fit of the model with the observed data was assessed using the Hosmer and Lemeshow goodness of fit test [35]. ANOVA analysis was performed to assess the effect of the experimental factors and their combination on the cannibalism rate. All analyses were performed using the software R version 3.0.2 [36].

## Results

### Simulations

The simulation results showed that the estimated number of encounters between larvae differed among the three containers. The tall and thin container showed the highest number of encounters between cannibal and victims, followed by the intermediate and the low and wide containers (Table 1). On the contrary, the space available for the victim to escape when encounters a cannibal (*f*) was the lowest in the low and wide container, followed by the tall and thin and intermediate containers (Table 1).

**Table 1:** Simulation results. The Unweighted (N) and weighted number of encounters (N*p*) between cannibal and victims are shown for each container. C.I., 95% Confidence Intervals; *f* is the space available for the victim to escape when encounters a cannibal.

	Container	Unweighted number of encounters (N) (95% C.I.)	*f* (mean ± sd)	Weighted number of encounters (N*p*) (95% C.I.)
Viewrange = 4				
	Tall and thin	8,354 (8,175; 8,534)	0.50 (0.05)	4,154 (4,028; 4,281)
	Intermediate	2,146 (2,056; 2,237)	0.58 (0.03)	896 (838; 955)
	Low and wide	1,342 (1,271; 1,414)	0.37 (0.01)	843 (787; 900)
Viewrange = 5				
	Tall and thin	10,968 (10,763; 11,174)	0.50 (0.06)	5,472 (5,327; 5,617)
	Intermediate	3,317 (3,205; 3,430)	0.58 (0.04)	1,378 (1,306; 1,451)
	Low and wide	1,857 (1,773; 1,942)	0.30 (0.01)	1,306 (1,236; 1,377)
Viewrange = 6				
	Tall and thin	13,524 (13,297; 13,752)	0.51 (0.07)	6,740 (6,580; 6,901)
	Intermediate	4,472 (4,341; 4,604)	0.59 (0.04)	1,855 (1,771; 1,940)
	Low and wide	2,405 (2,309; 2,502)	0.25 (0.01)	1,810 (1,727; 1,894)

The tall and thin container showed the highest number of encounters weighted for the likelihood of successful cannibalism (N*p*), while similar values were observed between the intermediate and the low and wide containers. The same pattern was observed using different values of *viewrange* (Table 1).

### Cannibalism experiments

In the control tests no missing larvae were found after 24 and 48 hours. In the test trials instead, L1 larvae disappearance was observed under all experimental conditions, while no L3 and L4 larvae disappearance was observed.

**Fig 1:**
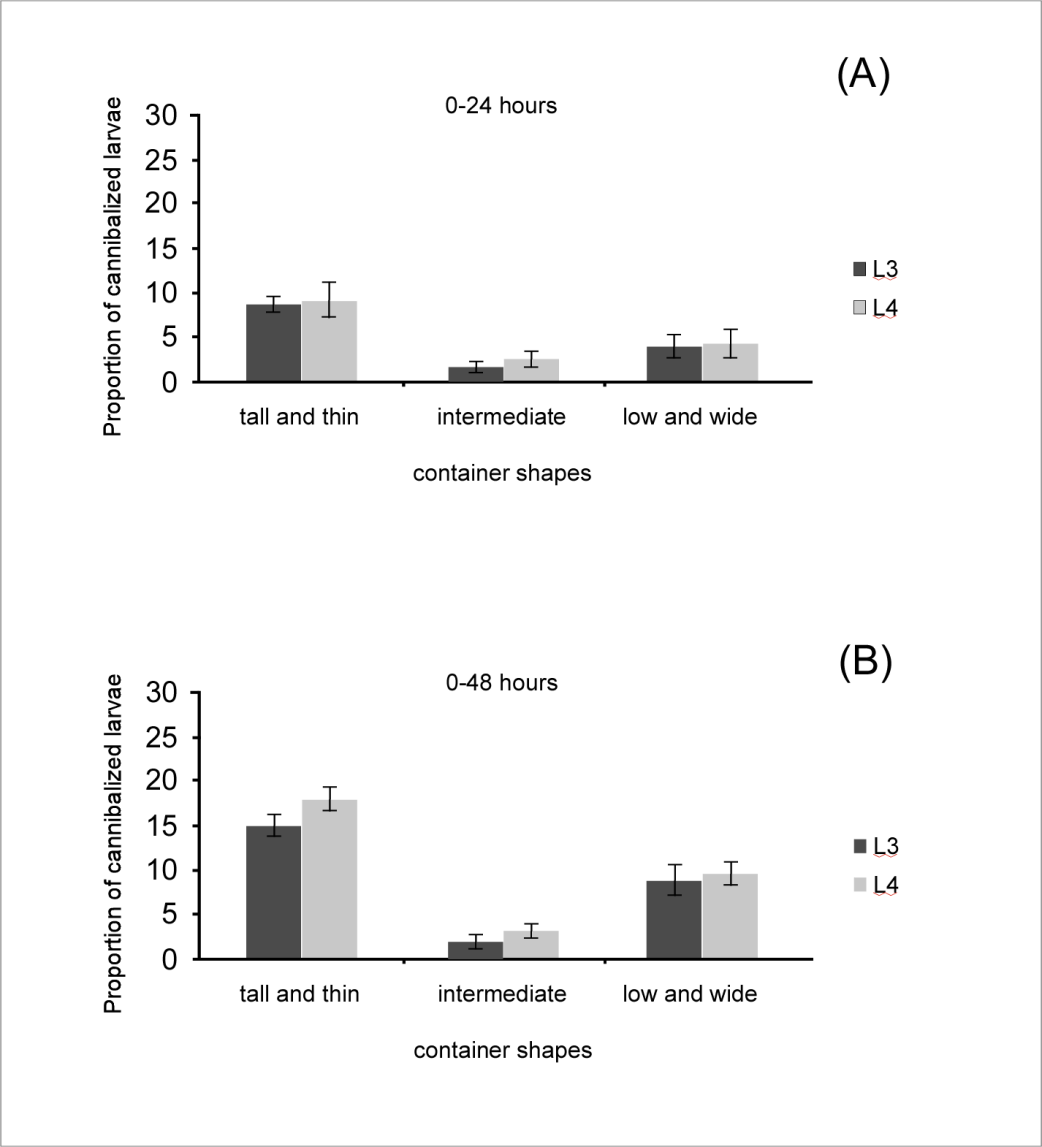
Cannibalism rate between *Aedes albopictus* larvae. The proportion of the cannibalized first-instar larvae is shown for each container shape. The values are shown as mean ± standard deviations. L3: third-instar larvae; L4: fourth-instar larvae.

In the experimental trials between L1 and L3 larvae, a percentage equal to 8.67±0.76%, and 15.33 ±1.44% of cannibalized larvae (i.e. missing larvae out of all larvae used in the experiment) were observed in the tall and thin containers after 24 and 48 hours (mean ± standard deviation), respectively. In the intermediate containers, the percentages of missing larvae after 24 and 48 hours were 1.67±0.29%, and 2.50 ±1.53%, while in those low and wide were 4.01±1.5%, and 9.33 ±2.52% (Fig 1).

In the experimental trials between L1 and L4 larvae, a percentage equal to 9.03±2.78%, and 18.17 ±1.53% were observed in the tall and thin containers after 24 and 48 hours, respectively. In the intermediate containers the percentages of missing larvae after 24 and 48 hours were 2.67±0.76%, and 3.80 ±1.53%, while in those low and wide were 4.33±2.02%, and 9.83 ±1.61% (Fig 1).

The Hosmer and Lemeshow goodness of fit (GOF) test showed a good fit of the data with the regression logistic model (χ^2^ = 0.4233, df = 8, *P*-value = 0.99). The ANOVA analysis showed that cannibalism rate was not affected by the larval instar as no significant differences were observed by comparing cannibalism rate between (L3-L1) and (L4-L1) trials in any containers (Table 2; Fig 1). Likewise, the length of contact between old and young larvae (24 and 48 hours) affected the cannibalism rate only in the tall and thin container (Table 2; Fig 1). On the contrary, the container shape significantly affected cannibalism rate in both (L3-L1) and (L4-L1) trials, with significantly higher cannibalism rate observed in the tall and thin containers than in the other two containers (Fig 1).

**Table 2:** ANOVA analysis performed for the cannibalism data in *Aedes albopictus*.

	Df	Deviance Resid.	Df Resid.	Dev	Pr(>Chi)
Null			359	557.66	
Instar	1	1.873	358	555.79	0.1711
Shape	2	130.419	356	425.37	2e-6***
Time	1	0.515	355	424.86	0.4729
Instar ×Shape× Time	2	1.077	348	414.45	0.5837
Instar ×Shape	2	1.028	353	423.83	0.5981
Instar × Time	1	0.105	352	423.72	0.7454
Shape× Time	2	8.198	350	415.52	**0.0166** *

*P< 0.05

***P< 0.001

## Discussion

### Cannibalism between old and young larvae

In this paper we found evidence that *Aedes albopictus* L3 and L4 larvae cannibalize L1 larvae. Under all experimental conditions, indeed, the disappearance of L1 larvae was observed, ranging from 2.5±0.5% to 18.17±1.5% after 48 h (Fig 1). We can confidently assume that the missing L1 larvae were cannibalized by older larvae as i) cannibalism between L1 larvae can be excluded because in the control tests, where no L3 and L4 larvae were introduced into the experimental chamber, no L1 disappearance was observed. Likewise, decomposition of the L1 larvae can also be excluded, because in the control tests where L1 dead larvae were used, no missing individual were observed after 48 hours; ii) the consumption of L1 larvae by L3 and L4 larvae has been described in other mosquito species and is in accordance with the expectation that cannibalism mostly occurs between older, bigger individuals and the younger [5,18–23].

*Ae*. *albopictus* is a vector species able to transmit several pathogenic viruses to humans, such as dengue, chikungunya and zika virus [29]. The findings of the consumption of young L1 larvae by the late instars can be particularly interesting from the epidemiological point of view. Cannibalistic behaviour, by affecting the number of individuals emerging from a breeding site could significantly affect the vector capacity of this species. On one hand, by limiting the number of larvae in the breeding sites it could maintain the population size below the carrying capacity, allowing it to grow. Furthermore, cannibalism, by offering a nutritional benefit to cannibal, can lead to the emerging of larger adult females with high fitness, high flight performance, host-seeking ability and dispersal potential, all characteristics affecting vector capacity [37–39]. On the other hand, however, cannibalism could reduce the number of emerging adults, and thus negatively impact on vector capacity [40,41]. Notably, our results, by showing that cannibalism rate is different between the experimental containers accordingly to their shape, suggest that the breeding sites can differently carry weight on vector capacity, and that the container shape plays a major role in generating this difference.

Cannibalistic behaviour between old and young larvae, could also affect the female oviposition behaviour and thus the distribution of mosquito vectors. Gravid females are highly selective in choosing oviposition sites, as immature stages are unable to move to a more suitable habitat if conditions become adverse [12]. In some mosquito species, including *Ae*. *albopictus*, the presence of conspecific larvae at low density is attractive for females, as it would indicate the site’s suitability (i.e. absence of predators and abundance of food resources). On the contrary, a high density of conspecific larvae has a repellent effect, because it would be a hallmark of intraspecific competition [42,43]. Our results showing cannibalism between late- and young-instars larvae in *Ae*. *albopictus* suggest that cannibalism can contribute to repel the oviposition of gravid females. The reduced hatchability of eggs observed in the presence of third-instar larvae in some species where cannibalism between old and young larvae occurs, such as *Anopheles gambiae* s.s. [44–46], would support this hypothesis. In *Ae*. *albopictus*, no studies specifically addressed if some oviposition behaviours evolved in response to cannibalism. Complex oviposition behaviours of females have been described, such as the scatter oviposition strategy, where egg batches are splitted between different breeding sites, and the asynchronous hatching of eggs [47]. Could be these strategies useful to avoid or reduce cannibalism between larvae? Or they are just an adaptation to limit larval competition? By affecting the larval density of the breeding sites, we could hypothesize that they can actually have some influence in these density-dependant interactions. Future studies specifically addressing these issues will allow to answer these questions.

### Container shape and cannibalism rate

The role of spatial shape on the cannibalism rate between late (L3 and L4) and young (L1) larval instars of the tiger mosquito *Ae*. *albopictus* was investigated using both experimental and simulation approaches. Simulation results showed that a different number of encounters between cannibal and victims characterized the three containers, with the highest values observed in the tall and thin containers (Table 1). If the cannibalism rate is affected by the number of encounters between cannibals and victims, we expected that the cannibalism rate observed in our experimental trials would be higher in tall and thin containers. Our results were concordant with this prediction showing that in all trials between old and young larvae, the rate of cannibalism significantly differed among the three container shapes, and was the highest within the tall and thin container (Table 1). Therefore, empirical and simulation results jointly suggested that container shape significantly affects the cannibalism rate by affecting the probability of encounter between individuals.

Empirical results showed that after the tall and thin container, the rate of cannibalism was higher in the low and wide than in the intermediate container, contrary to what we had been led to expect by simulations results, which predicted a lower number of encounters within the low and wide than in the intermediate container. However, the two containers showed a similar weighted number of encounters, and the low and wide container had also the lowest values in the *f* parameter (i.e. the space available for the victim to escape when encounters a cannibal) (Table 1). Taken together these results could explain the higher cannibalism rate observed in the low and wide container by suggesting the occurrence of encounters in more “favorable” positions for the cannibal, which could balance the effect of a lower number of encounters. According to our simulation and experimental results, therefore, the container shape would affect cannibalism not only by affecting the number of encounters between cannibal and victims but also by affecting the number of “favorable” encounters for the cannibal.

### Should we consider cannibalism as being affected only by the probability of encounter between larvae?

In the mosquito *Anopheles stephensi*, it has been suggested that the cannibalism observed between fourth- and first-instar larvae could arise from circular currents created by the filtering action of mouth brushes of fourth-instar larvae sweeping up first-instar larvae into the mouth parts. The cannibalism rate has therefore been considered to be the final product of random contacts between cannibal and victims [18]. However, mosquito larvae are able to actively attack co-specifics if close to one another as well as to escape from attacks [12,48]. Further ethological studies are needed to assess whether the eating of a co-specific is a by-product of the filtering actions or the consequence of voluntary attacks. Under both hypotheses, however, the probability of cannibal and victims encountering each other is a key factor in determining cannibalism rate. According to our results, the container shape plays a role by affecting the number and the propitiousness of encounters between cannibal and victims. Future studies should be performed to assess the interactions between the spatial shape and the other known factors affecting cannibalism, such as food or density. For example, we could hypothesize that the scarcity of food can lead to an increased foraging activity promoting the encounters between larvae, and thus cannibalistic events.

## Conclusions

To our best knowledge, our results represent the first evidence of larval cannibalism in the tiger mosquito *Aedes albopictus*. Temporary waters are inhabited by the immature stages of mosquitoes as well as many other arthropod species. Cannibalism at larval stage has been documented in several of them such as caddisflies [49], damselflies [50] and blowflies [51], and it has been shown to significantly affect both population demography and dynamics. In some cases, cannibalism has been suggested to be primarily due to the probability of encountering a vulnerable individual due to spatial topology. For example, in the pit-building antlion *Myrmeleon hyalinus* larvae, increased sand depth led to decreased cannibalism rate by providing a potential refuge from cannibals and reducing the encounters between cannibal and victims [52]. Our results, showing the role of spatial shape on cannibalism rates in mosquito larvae suggest that it can be a critical factor affecting the interactions occurring in this habitat, and highlight the importance of further study of its contribution in aquatic insects.

## Supporting information

S1 Appendix Larval movements. Observations of the time spent by the *Aedes albopictus* larvae at the top and the bottom in each container.
(DOCX)
