# Supplementary material for: Cannibalism in temporary waters: Simulations and laboratory experiments revealed the role of spatial shape in the mosquito Aedes albopictus
Source: PLoS One. 2018 May 29;13(5):e0198194. doi: 10.1371/journal.pone.0198194 (PMC5973580; doi:10.1371/journal.pone.0198194)
Supplement: S1 Appendix — Observations of the time spent by the Aedes albopictus larvae at the top and the bottom in each container. (DOCX) [file pone.0198194.s001.docx]

**S1 Appendix**

Individual first-, third- and fourth-instar larvae of *Aedes albopictus* were placed separately in the experimental containers filled with 200 ml of water and maintained under the same conditions of the cannibalism trials. The time spent by each larva at the top and the bottom was than registered during 10 minutes. Five larvae of each instar were used. The table below shows the observed results.

**Table S1.** Time spent by *Aedes albopictus* larvae at the top/bottom in the experimental containers. The values are shown as mean± standard deviation.

| **Container shape** | **Larval instar** |  | **Time spent in the top/bottom**  **(minutes)** |
| --- | --- | --- | --- |
|  |  |  |  |
| Tall and thin |  |  |  |
|  | L1 |  | 5.58±1.11 |
|  | L3 |  | 5.22±1.64 |
|  | L4 |  | 6.06±0.51 |
|  |  |  |  |
| Intermediate |  |  |  |
|  | L1 |  | 5.27±1.44 |
|  | L3 |  | 6.61±1.38 |
|  | L4 |  | 4.72±2.12 |
|  |  |  |  |
| Low and wide |  |  |  |
|  | L1 |  | 5.72±0.49 |
|  | L3 |  | 6.91±1.32 |
|  | L4 |  | 7.35±0.52 |
